# Production of Fatty Acids and Protein by *Nannochloropsis* in Flat-Plate Photobioreactors

**DOI:** 10.1371/journal.pone.0170440

**Published:** 2017-01-19

**Authors:** Chris J. Hulatt, René H. Wijffels, Sylvie Bolla, Viswanath Kiron

**Affiliations:** 1 Faculty of Biosciences and Aquaculture, Nord University, Bodø, Norway; 2 Bioprocess Engineering, AlgaePARC, Wageningen University, Wageningen, The Netherlands; Louisiana State University Health Sciences Center, UNITED STATES

## Abstract

*Nannochloropsis* is an industrially-promising microalga that may be cultivated for alternative sources of nutrition due to its high productivity, protein content and lipid composition. We studied the growth and biochemical profile of *Nannochloropsis* 211/78 (CCAP) in optimized flat-plate photobioreactors. Eighteen cultivations were performed at two nutrient concentrations. The fatty acid, protein content and calorific values were analyzed after 8, 12 and 16 days. Neutral lipids were separated and the changes in fatty acids in triglycerides (TAGs) during nutrient depletion were recorded. The maximum cell density reached 4.7 g∙L^-1^ and the maximum productivity was 0.51 g∙L^-1^∙d^-1^. During nutrient-replete conditions, eicosapentaneoic acid (EPA) and total protein concentrations measured 4.2–4.9% and 50–55% of the dry mass, respectively. Nutrient starvation induced the accumulation of fatty acids up to 28.3% of the cell dry weight, largely due to the incorporation of C16:0 and C16:1*n*-7 fatty acyl chains into neutral lipids. During nutrient starvation the total EPA content did not detectibly change, but up to 37% was transferred from polar membrane lipids to the neutral lipid fraction.

## Introduction

Sustainable, healthy diets for humans and animals may benefit from incorporating larger proportions of plant-based materials [1,2]. Phototrophic microalgae are an especially promising source of alternative food and feed ingredients, because many species of microalgae are able to synthesize additional metabolites that are not available from natural terrestrial plant sources [3–5]. As single-celled molecular factories, microalgae can also be cultivated on marginal land unsuitable for agriculture, using waste streams or saline water supplies [6,7].

At present, world aquaculture production especially is dependent on feed products from capture fisheries, and there is a need to find substitute materials that reduce the environmental costs [8,9]. Replacing feed ingredients with single-cell oils and proteins from microalgae could reduce the environmental impacts of aquaculture, improve the nutritional quality and reduce risks from pollutants that can accumulate in marine food chains [10–12].

Some species of microalgae synthesize very long chain fatty acids (carbon chains 20+ in length), including eicosapentaenoic acid (EPA, C20:5*n*-3) and docosahexaenoic acid (DHA, C22:6*n*-3) [13,14]. These omega-3 (*ω*-3) fatty acids are essential components of high quality diets for farmed fish and humans, and must be available in the correct amounts [5]. Fatty acids are the building blocks of lipids, but they are not distributed equally amongst different lipid classes. Polar algal lipids are located in structural and functional cell membranes, whilst neutral lipid triacylglycerols (TAGs) function as storage molecules [15]. Depletion of inorganic nitrogen and phosphorus in the growth medium can induce the accumulation of neutral lipids in oleaginous algae, but also initiates cell remodeling processes whereby membrane lipids are broken down and their constituent fatty acids are rebuilt into TAG. This redistribution of fatty acids amongst the different types of lipids could impact the bioavailability of fatty acyl chains when microalgae oils are incorporated into food and feed [16].

Nitrogen starvation also reduces the cell protein content and ultimately leads to the cessation of growth [17,18]. Therefore, to produce quality microalgal biomass as a whole-feed ingredient, cultivation techniques should aim to balance the lipid profile and the protein content. An alternative and potentially more efficient approach is a biorefinery-type system where microalgal oils could be separated from the cell biomass and used as concentrated feed or food supplements [19]. In this latter case, oil production could be maximized, nitrogen consumption minimized, and the residual biomass used for other processes including energy production [20,21].

Enclosed photobioreactors offer the highest levels of experimental control for developing optimal microalgae production systems [22]. Photobioreactors include various designs that can be broadly grouped into tubular systems [23,24], flat plate [25,26], column [27] and biofilm [28] configurations. Flat-plate photobioreactors with short light path lengths are amongst the best designs, because they have high volumetric efficiency (they have a high surface area to volume ratio) and consume less energy than tubular systems [29,30].

*Nannochloropsis* is a genus of robust, oleaginous microalgae that synthesizes EPA during balanced growth, and is a promising candidate for commercial applications [31–34]. In this study, we examine changes in the biochemical composition of *Nannochloropsis* sp. cultivated in optimized flat-plate photobioreactors as a potential feedstock for aquafeeds. We present the productivity, protein content and the lipid composition, including partitioning of LC-PUFAs into neutral lipids.

## Methods

### Cultivation

**Fig 1:**
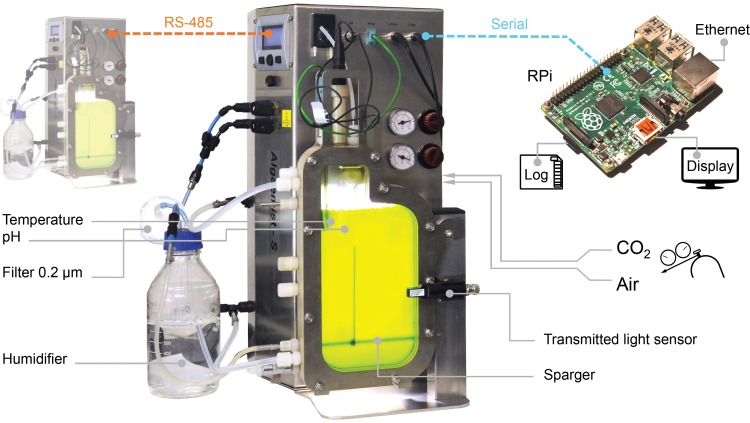
Configuration of the flat-plate photobioreactor systems. Photobioreactors used a 14 mm light path length with illumination by warm-white LED lights. Photobioreactors were set up, monitored and data-logged using a custom program running on a Linux single board computer.

The microalga *Nannochloropsis* sp. (strain 211/78, Culture Collection of Algae and Protozoa, United Kingdom) was cultivated in a pair of flat-plate photobioreactor systems (*Algaemist-S*, Ontwikkelwerkplaats, Wageningen UR, The Netherlands), illustrated in Fig 1. The bioreactor light path measured 14 mm and the total cultivation volume was 400 mL. The sparger and baffle were arranged in a draft-loop configuration (Fig 1). Cultures were sparged at 400 mL∙min^-1^ with 0.2 μm filtered air (Acrodisc® PTFE filters, Pall Corporation, USA) containing 1% CO_2_. The superficial gas velocity in the riser was 5.3×10^−3^ m∙s^-1^, and for the riser-downcomer combination the velocity was 3.7×10^−3^ m∙s^-1^. Light was provided by warm-white LEDs and a 16:8 hour light:dark photoperiod was used. Irradiance was calibrated with an Li-189 2π quantum sensor (Li-Cor, UK). The photon flux density on the front surface of the bioreactor was 180 μmol∙m^-2^∙s^-1^, although it was reduced to 90 μmol∙m^-2^∙s^-1^ for the first two diel cycles. The cultivation temperature was 25 ± 0.3°C, which was controlled with inbuilt heating and external cooling (F25, Julabo, Germany) systems. The culture medium was adjusted to an initial pH of 8.0 using NaOH and the reactor cultivation vessel was sterilized by autoclave (121°C, 20 min). Cultivation parameters were recorded every 5 minutes by a program written in Python (v2.7) and executed on a Linux-based single-board computer (Raspberry Pi, Raspberry Pi Foundation, United Kingdom). Recorded parameters included the incident photon flux density, transmitted photon flux, temperature, pH, CO_2_ flow and air flow. Seawater from Saltfjorden (Bodø, Norway) that was used for cultivation was filtered (~1.0 μm glass-fibre, VWR, Norway), aged for a week, and then filtered again through 0.1 μm Durapore^®^ membranes (Millipore, USA). The nutrient medium was f/2 formulation [35] with concentrations rescaled to support high biomass densities. Two different nitrate and phosphate (NP) concentrations were used: (i) 1.5 g∙L^-1^ NaNO_3_ + 0.1 g∙L^-1^ NaH_2_PO_4_ *vs* (ii) 3.0 g∙L^-1^ NaNO_3_ + 0.2 g∙L^-1^ NaH_2_PO_4_, referred to as low-NP and high-NP respectively (S1 Table).

### Experimental design

*Nannochloropsis* was cultivated for 8, 12 or 16 days in high-NP and low-NP medium. Each treatment was replicated three times (*n* = 18 cultivations) completing a 3×2 design. Experiments were conducted using two photobioreactor units and the repetition sequence mitigated any systematic combination of treatment, individual bioreactor, and time (S2 Table).

### Biomass measurements

Samples of cultivation broth (0.5–1.5 mL) were collected daily to measure the absorbance at 540 and 680 nm in a 1 cm micro-cuvette [36], using a spectrophotometer (Hach-Lange DR3900, Hach, International). The samples were diluted with fresh medium (1 to 25 fold) using calibrated micropipettes so that the absorbance was within the range 0.4–1.0 units for linear response. The dry-weight of each sample was measured at the end of each batch culture by filtering 5–10 mL of broth through weighed 1.0 μm 47 mm glass-fiber filters (VWR). Filters were rinsed with isotonic ammonium formate (0.5 M) to remove extracellular salt, dried at 95°C for 48 h and re-weighed. The dry-weight was approximately linearly related to the absorbance at 540 nm (S1 Fig) and derived as in Eq 1.

$$W=\left(0.214∙A_{540}\right)+0.109$$ (1)

Where *W* is the dry weight (g∙L^-1^) and A_540_ is the absorbance at 540 nm. The absorbance ratio (A_680_/A_540_) of the cell suspension was calculated to indicate the ratio of chlorophyll (A_680_) relative to the biomass (A_540_). Samples for metabolite analysis was collected at the end of each batch culture at 8, 12 or 16 days by centrifugation (2000 rcf, 5 min). Samples were washed, pelleted and re-suspended three times with ammonium formate to remove salt, and then stored at -20°C.

### Nitrate analysis

Samples for nitrate analysis were centrifuged (2000 rcf, 5 min) and the supernatant was stored at -20°C. The concentration of nitrate in the broth was measured with standard colorimetric reagents using a miniaturized microplate method [37]. The conversion of nitrate to nitrite was performed using NADH:nitrate reductase (Nitrate Elimination Company, USA) and the absorbance was measured at 540 nm (FLUOstar Optima, BMG Labtech, USA). Seven-point linear calibrations were included in each plate with *r*^2^ values >0.996 (S2 Fig).

### Lipid analysis

Fatty acid concentrations were measured by gas chromatography (GC) of methyl-ester derivatives. Frozen sample pellets were lyophilized for 72 hours before the lipids were extracted and analyzed in duplicate. Approximately 8 mg of lyophilized biomass was weighed using a precision balance (MX5, Mettler-Toledo, USA) before the lipids were extracted and fatty acids prepared using the methods of Breuer et al. [38]. A bead mill (MagNA lyser, Roche, Switzerland, 0.1 mm glass beads) and sonicator (Elmasonic S-120, Elma Schmidbauer, Germany) were used to disrupt the cells and extract lipids. Lipid extracts were derivatized to fatty-acid methyl-esters (FAMEs) using 12% HCl in methanol and heated at 70°C for 3 h. FAMEs were separated and quantitated using a Scion 436 GC (Bruker, USA) fitted with a flame ionisation detector, a splitless injector and a DB-23 column (Agilent Technologies, USA). Supelco^®^ 37-component standards (Sigma-Aldrich, USA) were used for identification and quantitation of the FAMEs with five-point calibrations (0 to 0.4 mg∙mL^-1^, *r*^2^≥0.999, S3 Fig) [39]. Blanks were included in the extraction process to eliminate background trace peaks. Tripentadecanoate (Sigma-Aldrich, USA) was used as an internal standard to determine fatty acid recovery and transesterification efficiency. Derivatization adds a methyl group (+14 atomic mass units to a free fatty acid) and thus GC analysis of derivatives over-estimates the mass of fatty acids per unit biomass. Data was mass-corrected and the results are equivalent to mg of free fatty acid per gram dry cell weight. Fatty acids incorporated into neutral lipids were measured in the low-NP treatment samples. Lipid accumulation in high-NP samples was minimal and so neutral lipids were not studied in these samples. Neutral lipids were separated by solid-phase extraction using 6 mL volume, 1 gram silica cartridges (Supelco, USA). Total lipid extracts were loaded onto columns and the neutral lipids were eluted with hexane:diethyl ether (7:1 v:v). The solvent was evaporated under nitrogen and the samples were derivatized as described previously.

### Calorific value

The calorific value of the biomass at the end of each cultivation (*n* = 18) was measured with an oxygen bomb calorimeter (C200, IKA, Germany). The instrument was calibrated with benzoic acid standards with relative standard deviation ± 0.23% (*n* = 5).

### Total protein

The total protein content of lyophilized cells was determined with the Lowry method using a BioRad-DC Protein Assay kit (BioRad, USA) according to the manufacturers recommended procedures. Samples were weighed as described above, then prepared by bead milling in lysis buffer (60 mM Tris, 2% sodium dodecyl sulfate). Ten-point standard curves (r^2^ >0.996, S4 Fig) were used and samples were analyzed in duplicate.

### Cell size

The sizes of approximately 3 to 5×10^5^ cells were measured with a Coulter Multisizer 3 (Beckman Coulter, International) fitted with a 50 μm aperture. Duplicate samples were measured from each experimental replicate and the size-frequency distributions were blank-corrected.

### Growth curve fitting

To describe the growth trajectory, productivity and specific growth rate, a subset of cultures maintained for 16 days (3 high-NP, 3 low-NP) were modeled using a 4-parameter logistic function (Eq 2), which captures the lag, exponential and stationary phases in a single model.

$$C_{X}=\phi 1+\frac{\phi 2-\phi 1}{1+\exp\left(\frac{\phi 3-t}{\phi 4}\right)}$$ (2)

Where C_*X*_ is the dry weight (g∙L^-1^) at time *t* (days), *ϕ*1 is the lower asymptote (minimum C_*X*_), *ϕ*2 is the upper asymptote (maximum C_*X*_), *ϕ*3 is *t* at 0.5ϕ2 (the inflection point, the time of maximum growth) and *ϕ*4 is the scale parameter [40]. Modeling the growth trajectory allows us to extend the data to accurately estimate the productivity over time (Eq 3).

$$P_{i}=\frac{C_{X,i}-C_{X,i-1}}{t_{i}-t_{i-1}}$$ (3)

Where *P*_*i*_ is the productivity (g∙L^-1^∙d^-1^) at time *t*_*i*_, C_*X*,*i*_ is the dry weight (g∙L^-1^) at the *i*^th^ time, and a 1 hour time step was selected. The maximum value (P_max_) was recorded. The specific growth rate of biomass in the photobioreactor, *k* (d^-1^), was subsequently derived (Eq 4).

$$k_{i}=\frac{P_{i}}{C_{X,1}}$$ (4)

The maximum biomass yield on light (Y_X/mol_, g biomass per mol PAR) was calculated (Eq 5).

$$Y_{X/mol}=\frac{P_{max}}{I_{mol}}$$ (5)

Where P_max_ is the maximum productivity (g∙L^-1^∙d^-1^) and I_mol_ is the photon flux received by 1 L of broth in a photobioreactor each day (mol∙L^-1^∙d^-1^). Data analysis was conducted using the R programming language [41].

## Results

### Growth and cultivation conditions

**Fig 2:**
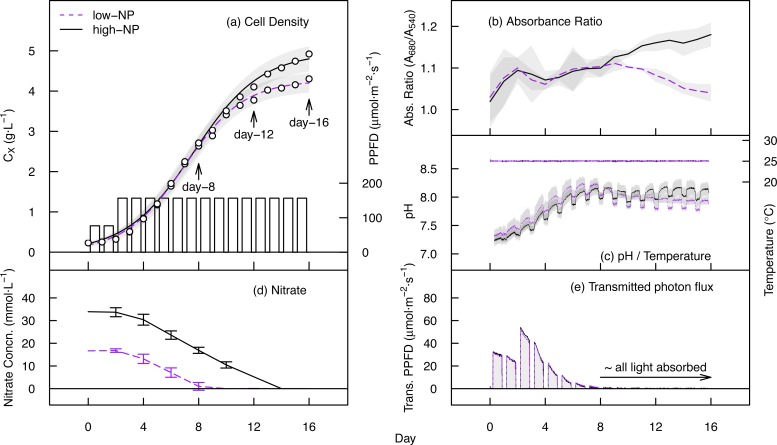
Cultivation data for *Nannochloropsis* in flat-plate photobioreactors. (a) Growth trajectory and irradiance pattern of 16-day cultivations in high-NP and low–NP nutrient treatments. Fitted lines are from the logistic model and shaded areas indicate the standard error of the fitted values. (b) Absorbance ratio (A_680_/A_540_) of the cell suspension. (c) Broth pH and temperature. (d) Concentration of extracellular nitrate (mM∙L^-1^). (e) Transmitted photon flux is the photon flux exiting the rear face of the reactor.

The growth trajectories and cultivation parameters for the 16-day cultivations of *Nannochloropsis* are shown in Fig 2. Cultivations maintained for shorter 8 and 12 day periods followed the same patterns, and are provided in S5 Fig. After 16 days of cultivation, the cell density attained in the low-NP and high-NP treatments measured 4.2 ± 0.9 and 4.7 ± 0.3 g∙L^-1^ respectively (Table 1). In the low-NP treatment, nitrate was eliminated from the broth by day 8, whilst in the high-NP treatment nitrate was exhausted by day 14 (Fig 2D). The absorbance ratio (A_680_/A_540_) peaked at 1.11 in the low-NP treatment after 9 days of cultivation before declining to 1.04 at 16 days, indicating the loss of chlorophyll *a* (chlorosis) during nutrient starvation. In the high-NP treatment, the absorbance ratio continued to increase, reaching a maximum of 1.18 at the end of the cultivation period. This was related to decreasing average light intensity within the cultures during growth. The pH increased from approximately 7.3 ± 0.2 to 8.1 ± 0.2 during nutrient uptake in the first eight days (Fig 2C), with short-term fluctuations reflecting changing photosynthetic CO_2_ uptake during illuminated/dark periods. The photon-flux exiting the rear face of the reactor was <1.0 μmol∙m^-2^∙s^-1^ beyond day 8, showing that almost all of the incident light was absorbed after this time (Fig 2E).

**Table 1:** Biomass properties of *Nannochloropsis* sp. cultivated in flat plate photobioreactors.

Treatment	Day	Dry weight (g∙L^-1^)	CV (kJ∙g^-1^)	Total Protein (%)	Cell dia. (μm)
high-NP	8	2.37 (0.77)	21.75 (1.47)	52.2 (4.0)	2.75 (0.15)
high-NP	12	3.81 (0.35)	22.90 (0.48)	54.9 (1.7)	2.81 (0.20)
high-NP	16	4.68 (0.32)	23.52 (0.51)	49.6 (6.8)	2.72 (0.07)
low-NP	8	2.44 (0.37)	22.98 (0.48)	49.9 (2.0)	2.81 (0.16)
low-NP	12	3.52 (0.16)	24.61 (1.48)	31.1 (2.6)	2.64 (0.07)
low-NP	16	4.21 (0.87)	25.68 (0.53)	24.6 (1.5)	2.64 (0.05)

Values are mean (± *SD*) of *n* = 3 cultivations per treatment.

**Fig 3:**
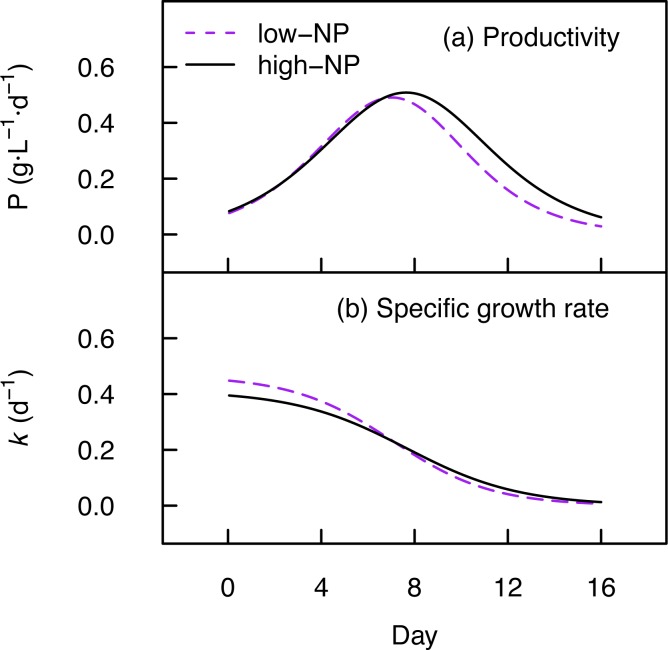
Growth performance of *Nannochloropsis*. (a) The productivity (P) was computed from the growth curves (C_X_, Fig 2A) with a 1 hour timestep. (b) The specific growth rate (*k*) was then calculated from the biomass concentration (C_X_) and productivity (P) over time. Lines are the treatment means.

The productivity (P) and specific growth rate (*k*) computed from the logistic models are illustrated in Fig 3. The maximum productivity (P_max_) in low-NP and high-NP treatments was 0.50 and 0.51 g∙L^-1^∙d^-1^ dry biomass at 6.6 and 7.5 days (Table 2) and the maximum specific growth rate (*k*_max_) was 0.45 ± 0.05 and 0.40 ± 0.03 in low-NP and high-NP treatments respectively. Accounting for the photoperiod, the reactor frontal area and its volume, the maximum biomass yield on light was 0.68 and 0.70 g∙mol^-1^ photons (PAR) in low-NP and high-NP treatments, respectively.

**Table 2:** Summary statistics of *Nannochloropsis* growth in low-NP and high-NP medium.

Treatment	P_max_	*k*_max_	PE (g∙mol^-1^)
low-NP	0.50 (0.01)	0.45 (0.05)	0.68 (0.01)
high-NP	0.51 (0.03)	0.40 (0.03)	0.70 (0.04)

Variables are calculated from the maximum productivity (Fig 3) and presented as mean ± *SD* (*n* = 3).

### Cell size

The cell size of *Nannochloropsis* sp. remained approximately the same amongst all conditions. A two-way ANOVA indicated no detectable effect of nutrient treatment (*F* = 0.96, p = .343) or sampling time (*F* = 1.75, p = .207) on the mean cell diameter (S3 Table, S6 Fig). Across all treatments, the mean diameter of *Nannochloropsis* sp. measured 2.73 ± 0.13 μm.

### Total fatty acids

The fatty acids that were detected and resolved to isomer level were; C14:0, C16:0, C16:1*n*-7, C18:0, C18:1*n*-9, C18:2*n*-6, C20:4*n*-6 (ARA), and C20:5*n*-3 (EPA). The fatty acid extraction and derivatization procedure led to high recovery and transesterification efficiency, measuring 97.7 ± 7.8%. The changes in fatty acid content during different growth stages of batch cultivation are shown in Table 3, where total fatty acids increased from 100 mg∙g^-1^ (10% of the dry weight) at day 8 to 281 mg∙g^-1^ (28.1% of the dry weight) at day 16 in the low-NP treatment. In comparison, high-NP cultivation yielded just 12.5% fatty acids at day 16. The dominant fatty acids present in nutrient-replete *Nannochloropsis* were C16:0, C16:1*n*-7 and C20:5*n*-3. Oleic acid (C18:1*n*-9) typically remained at low abundance (~0.1% cell dry weight) during nutrient-replete growth, but co-accumulated with C16:0 and C16:1*n*-7 in the stationary phase up to 2.8% of the cell dry weight. Hexadecanoic acid (C16:0) dominated the fatty acid profile during nutrient-starved conditions, reaching 37% of total fatty acids in the low-NP treatment stationary phase (day 16). The total amount of PUFAs increased only slightly in response to nutrient starvation; in the low-NP treatment PUFAs measured 45 mg∙g^-1^ (45.4% total fatty acids) at day 8, but increased to 61 mg∙g^-1^ (21.8% total fatty acids) at day 16. In contrast, saturated fatty acids increased substantially from 30 mg∙g^-1^ to 122 mg∙g^-1^ over the same period. The total EPA content varied from 41.5 to 46.8 mg∙g^-1^. The highest percent share of EPA was 45.7% total fatty acids (high-NP, day 8) and the lowest percent share of EPA was 16.7% total fatty acids (low-NP, day 16). ARA was present at very low abundance during nutrient-replete cultivation, but accumulated to just over 0.5% of the dry mass (day 12 & 16, low-NP). The ratio of *ω*-3 to *ω*-6 fatty acids was high during nutrient-sufficient growth (day 8), largely driven by the abundance of EPA, but decreased during nutrient starvation (day 16, low-NP) due to the accumulation of C18:2*n*-6 and ARA (C20:4*n*-6).

**Table 3:** Changes in total fatty acids (mg∙g^-1^ dry weight) during different growth stages of *Nannochloropsis* cultivated at two NP concentrations.

	high—NP						low—NP					
	8		12		16		8		12		16	
C14:0	4.5	(0.7)	5.4	(0.6)	7.5	(0.9)	5.5	(0.4)	10.3	(0.9)	15.0	(1.9)
C16:0	19.4	(0.3)	21.6	(2.2)	31.0	(2.8)	23.6	(2.0)	62.5	(1.0)	103.7	(3.7)
C16:1*n*-7	21.8	(1.2)	23.0	(2.8)	28.3	(1.5)	23.6	(1.5)	43.1	(1.1)	69.2	(3.5)
C18:0	0.3	(0.4)	0.2	(0.2)	0.5	(0.8)	0.8	(1.6)	1.8	(0.8)	3.7	(0.5)
C18:1*n*-9	0.9	(1.5)	1.2	(1.9)	4.4	(1.3)	1.3	(2.1)	12.8	(1.2)	28.0	(1.9)
C18:2*n*-6	2.5	(1.2)	2.9	(2.5)	4.4	(2.0)	1.7	(1.9)	5.6	(0.6)	7.6	(0.7)
C20:4*n*-6	0.3	(0.3)	0.3	(0.3)	0.3	(0.3)	0.3	(0.3)	5.3	(2.0)	6.8	(0.1)
C20:5*n*-3	41.5	(2.7)	43.2	(3.6)	49.3	(3.9)	43.2	(1.1)	45.0	(5.1)	46.8	(2.4)
Σ SAT	24.0	(0.7)	27.0	(1.7)	38.8	(4.2)	29.9	(1.6)	74.6	(2.1)	122.4	(6.0)
Σ MUFA	22.5	(0.6)	23.9	(3.0)	32.7	(2.5)	24.6	(3.3)	55.9	(0.8)	97.2	(3.1)
Σ PUFA	44.4	(1.4)	46.4	(5.7)	53.9	(6.0)	45.2	(2.1)	55.9	(7.4)	61.2	(3.0)
Σ ω-3	41.5	(2.7)	43.2	(3.6)	49.3	(3.9)	43.2	(1.1)	45.0	(5.1)	46.8	(2.4)
Σ ω-6	2.8	(1.5)	3.2	(2.6)	4.7	(2.1)	2.0	(2.1)	10.9	(2.5)	14.5	(0.6)
ω-3/ω-6 Ratio^+^	14.6		13.4		10.5		21.3		4.1		3.2	
Total Fatty Acids	90.8	(0.7)	97.3	(10.1)	125.4	(10.9)	99.6	(6.4)	186.3	(4.6)	280.8	(6.0)

The time points correspond to the exponential (day 8), early stationary (day 12) and stationary phase (day 16) respectively. For each, the mean and (± *SD*) is given, *n* = 3.

^+^ Indicates the ratio of treatment means.

SAT- saturated fatty acids; MUFA- monounsaturated fatty acids; PUFA—polyunsaturated fatty acids

### Fatty acids in neutral lipids (TAG) of *Nannochloropsis*

**Fig 4:**
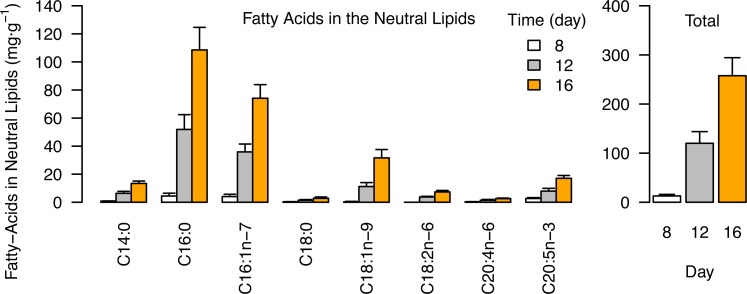
Fatty acids in the neutral lipids (TAG) of *Nannochloropsis*. (a) Individual fatty acids (b) Total fatty acids. Data are mg∙g^-1^ dry cell weight after 8, 12 and 16 days of cultivation in low-NP medium. Data is the mean of three replicate cultivations and error bars indicate the standard deviation.

The accumulation of fatty acids in the neutral lipids (~TAGs) during different growth phases in the low-NP treatments is presented in Fig 4. All fatty acyl chains in neutral lipids increased from day 8 to day 16, but were dominated primarily by C16:0 and C16:1*n*-7 fatty acids that together accounted for 71% of the total at day 16 (Fig 4, Table 4). In nutrient-replete conditions (day 8) the fatty acids in neutral lipids totaled just 13 ± 3 mg∙g^-1^ (1.3% of the cell dry weight), but increased to 258 ± 37 mg∙g^-1^ (25.8% cell dry weight) by day 16. PUFAs were under-represented in neutral lipids, EPA accounting for only 17 ± 0.7 mg∙g^-1^ (6.8% of the neutral lipid fatty acids) at day 16.

**Table 4:** Percentage fatty acid composition (% total fatty acids) of *Nannochloropsis* neutral lipids (TAG) at three different growth phases in low-NP medium.

Day	C14:0	C16:0	C16:1*n*-7	C18:0	C18:1*n*-9	C18:2*n*-6	C20:4*n*-6	C20:5*n*-3
8—exponential	6.7 (1.1)	33.3 (8.5)	30.7 (5.2)	2.6 (2.0)	1.4 (2.4)	0.0 (0.0)	2.8 (1.4)	22.5 (11.0)
12—early stationary	5.3 (0.1)	43.2 (0.2)	30.1 (1.1)	1.2 (0.1)	9.3 (0.5)	3.2 (0.3)	1.2 (0.3)	6.6 (0.5)
16—stationary	5.3 (0.3)	42.1 (0.6)	28.9 (0.6)	1.1 (0.2)	12.1 (0.8)	2.8 (0.3)	1.0 (0.2)	6.8 (0.4)

For each, the mean (± *SD*) is given, *n* = 3.

### Protein content

**Fig 5:**
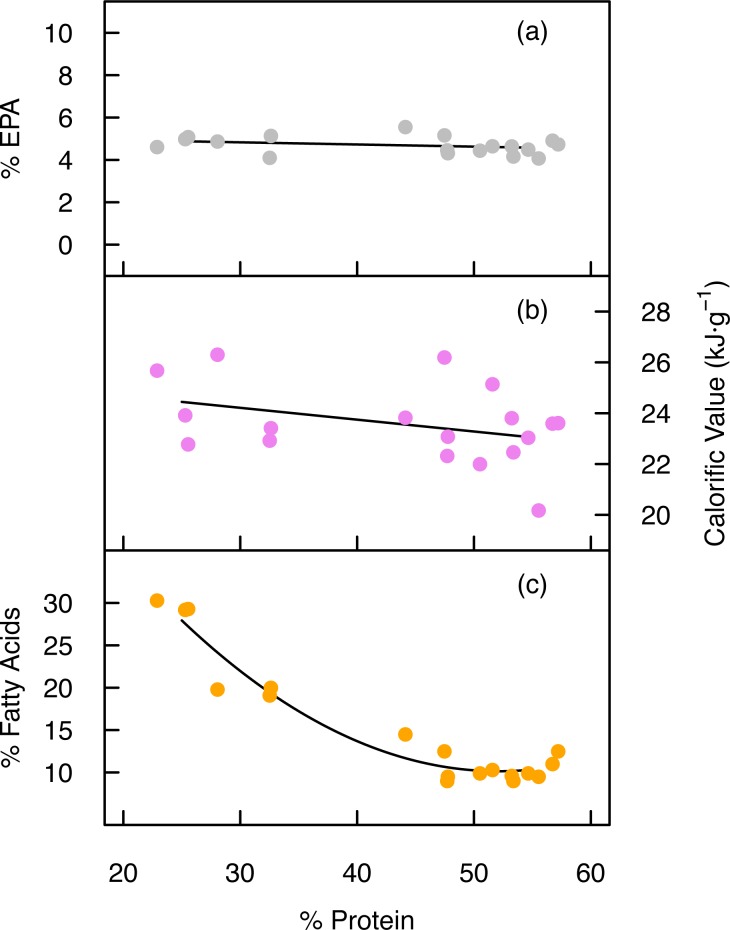
Cell protein content *vs* EPA, calorific value and total fatty acids. (a) The relationship between the percentage of protein and the percentage EPA in dry cell mass after 8, 12 and 16 days of cultivation in low-NP and high-NP treatments is described by a linear fit. (b) The relationship between the percentage of protein and the calorific value after 8, 12 and 16 days of cultivation in low-NP and high-NP treatments is described by a linear fit. (c) The relationship between the percentage protein and total fatty acids across all treatments is described by a quadratic fit (for visual guidance) where *y* = 0.024∙*x*^2^–2.52∙*x* + 75.9. Each data point is derived from a single cultivation (*n* = 18).

The highest protein content was attained during nutrient-replete growth, measuring 54.9 ± 1.7% of total dry mass, whilst the lowest protein content measured just 24.6 ± 1.5% (Table 1). Despite the substantial reduction in protein content in the low-NP treatment, linear regression analysis revealed that the EPA content of the cells remained unaffected (*F* = 1.66, p = .215, Fig 5A), indicating that the protein content could be adjusted without impacting the total amount of EPA in cells. The cell protein content was inversely related to the total fatty acid content however (Fig 5C).

### Calorific value

The calorific value increased following nutrient exhaustion and reduction of protein content (Fig 5B). The lowest calorific value was observed in the exponential phase after 8 days, measuring 23.0 ± 0.5 and 21.8 ± 1.5 kJ∙g^-1^ in low-NP and high-NP treatments respectively (Table 1). Cultivation to 16 days increased the calorific value to 25.7 ± 0.5 kJ∙g^-1^ in the low-NP treatment, with the high-NP treatment increasing to 23.5 ± 0.5 kJ∙g^-1^.

## Discussion

High cell densities and productivity are necessary for efficient industrial production of microalgal biomass. In the present study we were able to obtain a maximum dry mass of 4.7 g∙L^-1^ and productivity of 0.51 g∙L^-1^∙d^-1^ for *Nannochloropsis*. Nutrient starvation induced the accumulation of total fatty acids up to 28.1% of the dry cell weight and the partitioning of fatty acyl chains from polar to neutral lipids may be important for nutrition applications.

### EPA is conserved and partially transferred to neutral lipids (TAG) during nutrient starvation

Previous reports indicate that, based on percent fatty acid composition, a substantial reduction in the EPA content can occur in *Nannochloropsis* during nutrient starvation [42]. Our data shows that total EPA (mg∙g^-1^ dry weight) was conserved however, with no evidence of significant breakdown or synthesis of total cell EPA under nutrient starvation. The percent share of EPA was however reduced from 44.0% to 15.5% total fatty acids upon nutrient starvation in low-NP medium, which may impact the overall quality of oil products from this species. Furthermore, up to 37% of the total cell EPA was transferred from the polar lipids to the neutral lipid fraction during nutrient starvation (day 16, low-NP). The ability to increase the total lipid content and the calorific value of *Nannochloropsis* sp. without appreciably reducing the total EPA content does provide some flexibility for tailoring the total lipid content, but may also lead to changes in the oil quality from this species.

### Polyunsaturated fatty acid synthesis, precursors and intermediates

The *ω*-3/6 ratio measured up to 22.9 during nutrient-replete growth, but under nutrient starvation the ratio was reduced to 3.2. High *ω*-3/6 ratios are desirable for aquafeeds, but are difficult to achieve with natural crop-plants, which makes microalgae lipids potential substitutes for fish oil [5]. Long-chain PUFAs are synthesized by the *ω*-6 and/or *ω*-3 pathways in eustigmatophytes [43]:

[ω-6]:C18:2n-6→(Δ6-d)→C18:3n-6*→(e)→C20:3n-6*→(Δ5-d)→C20:4n-6→(Δ17-d)→C20:5n-3

[ω-3]:C18:2n-6→(Δ15-d)→C18:3n-3→(Δ6-d)→C18:4n-3→(e)→C20:4n-3→(Δ5-d)→C20:5n-3

Where *d* and *e* correspond to desaturase and elongase enzymes respectively. The *ω*-6 pathway is reportedly dominant [43] and the presence of ARA (C20:4*n*-6) in *Nannochloropsis* indicates that the intermediate metabolites (marked*) must also be synthesized. However, these were below the detection limit in our samples, and are expected to be rapidly metabolized.

Most farmed aquatic animals have dietary requirements for DHA, EPA, ARA, linoleic (C18:2*n*-6) and linolenic (C18:3*n*-3) acids [44]. Although C18:3*n*-3 was not present here in *Nannochloropsis*, C18:2*n*-6 accumulated to approximately 0.5% of the cell dry weight during the stationary phase. Freshwater fish are able to convert C18:2*n*-6 to long chain PUFAs with two desaturase enzyme systems (Δ^6^ and Δ^5^) and three elongases. Both freshwater and diadromous fish also require 3–10% of EPA from total fatty acids (1–7.5 g∙kg^-1^ total diet) [44], which could be satisfied by *Nannochloropsis* throughout growth, including the stationary phase. EPA in nutrient-replete *Nannochloropsis* is found predominantly in polar galactolipids and, although fish are known to utilize EPA in both TAG and phospholipids [45], little research has been conducted on the bioavailability of LC-PUFAs from photosynthetic microalgae sources.

### *Nannochloropsis* selectively accumulates C16:0 and C16:1 fatty acids in neutral lipids (TAG)

Neutral lipids in nutrient-starved microalgae can contain 90% or more TAG, but may also include free fatty acids and sterols [46–48]. Our data show that the increase in fatty acids during nutrient starvation was largely due to increases in saturated C16:0 and monounsaturated C16:1*n*-7 acyl chains that are typically incorporated into storage triacylglycerols (TAGs) [46]. Accumulation of these C16-series fatty acids in TAG is ideal for biodiesel production [49], but may be of limited nutritional and economic value as feed ingredients. The mechanisms of *de-novo* fatty acid biosynthesis and TAG assembly are broadly understood for many microalgae [43,34], but the regulatory networks involved in the degradation, inter-conversion and repackaging of LC-PUFAs such as EPA during stress conditions are not well described. These mechanisms could be important for determining the partitioning of *ω*-3 fatty acids into different lipid groups and thus altering their bioavailability in food and feed products. The lipid class and the *sn* position (the fatty acid position in TAG molecules) can impact the bioavailability of fatty acids in human foods [50], although a recent study found that EPA in polar lipids from *Nannochloropsis* can have similar bioavailability to that from Krill oil [16].

### Protein content

In nutrient-replete conditions, *Nannochloropsis* cell mass comprised 50–55% crude protein, which is a good value for a whole-cell ingredient in aquafeeds [51]. Generally, salmonids require 36–38% digestible protein (dry-matter basis) in their diets, but this reduces to 29% for tilapia, an omnivorous fish [51]. Conventional aquafeeds have traditionally relied heavily on protein from fishmeal, but improved modern feeds substitute a large amount of fish-derived ingredients for soy protein. Our upper protein values for whole-cell *Nannochloropsis* during exponential growth are slightly lower than fishmeal (62–72%), but higher than whole soybean meal (44%), although soy protein concentrates can contain 63% or more protein [51]. Our data compares well with the freshwater microalgae *Arthrospira* (58%) and *Chlorella* (52%) [52]. *Nannochloropsis* has also been tested in the diets of some farmed species in feeding trials. In a trial with Atlantic cod, feeding a combination of *Nannochloropsis* sp. and *Isochrysis* sp. (28% inclusion) reduced feed intake and growth significantly [53]. However, defatted biomass (the residual biomass after lipid extraction) of *Nannochloropsis* (up to 20% inclusion) was found to offer comparable growth rates to conventional aquafeeds in studies with Atlantic salmon [54]. *Nannochloropsis* is a small microalga with a tough cell wall structure comprised largely by cellulosic material [55]. Consequently, the bioavailability of cell proteins and lipids may be limited when whole-cell biomass is incorporated into diets. For complete cell disintegration, it was necessary in this work to use a combination of bead milling and ultrasound. The effective recovery of metabolites and the bioavailability of cell contents should however be considered in industrial applications. Bead milling can consume a large amount of energy [56,57], and so industrial methods for extraction of oils and proteins, for example, may need careful selection. The extraction efficiency of intracellular components also varies during the growth stage, with nitrogen depletion improving the release of intracellular components [18].

### Light energy conversion

Photobioreactors are devices that are optimized to convert light energy into biomass energy and specific products- they are ‘designer leaves’. High light energy conversion efficiency is vital for biofuels and will be equally important for sustainable food and feeds. Our photobioreactors are model experimental systems that use artificial LED light sources. Since photosynthesis is limited to a maximum of around 8–12% solar energy conversion [21], full scale production systems will need to use natural sunlight, but our lab-scale systems offer the opportunity for controlled experiments. In our simulated 16:8 hour light cycle (180 μmol∙m^-2^∙s^-1^ PAR during illumination), we obtained light conversion efficiency up to 0.70 g biomass per mol of PAR reaching the outermost surface of the broth. These values are comparable to other studies in optimized photobioreactor systems, e.g. 0.88 to 0.48 g∙mol^-1^ for *Chlamydomonas* under constant light at 100 to 500 μmol∙m^-2^∙s^-1^ [58].

The major energy cost in maintaining enclosed photobioreactor systems is that embodied in the supply of power for gas exchange and mixing. The absorbance of light for photosynthesis scales with the illuminated surface area of the photobioreactor (m^2^), whilst the energy embodied in mixing is proportional to the reactor volume (m^3^). Thus, flat plate photobioreactors that have short light path lengths (high surface area to volume ratios) are the most energetically efficient production platforms. Assuming that the CV of the biomass during maximum production was 21.75 kJ∙g^-1^ (high-NP, day 8), we can approximate a maximum gross daily bioenergy yield of 11.1 kJ∙L^-1^∙d^-1^ in our model system. The power supply measured 34 W∙m^-3^, which is equivalent to 2.96 kJ∙L^-1^∙d^-1^ ([59] and references therein). This is the amount of energy needed to pump gas through the broth, and there are additional efficiency costs in energy supply upstream. Therefore, our maximum energy return on investment (EROI) for sparging was approximately 3.75. This is not the bottom-line energy balance of algal production, but we introduce it as a useful metric for assessing photobioreactor performance, since other parameters either have less impact on cultivation performance, or are proportional to the growth rate (e.g. nutrient supply).

### Conclusions

For aqua-feeds, microalgae containing sufficient *ω*-3 fatty acids and protein are desired. Cultivation of *Nannochloropsis* in nutrient-replete conditions or subject to only mild nutrient depletion maintains the necessary protein content, and probably offers the best strategy for producing a complete whole-cell biomass for nutrition applications. Although *Nannochloropsis* is a strong candidate for providing algal feedstocks for nutrition markets, the biomass of several species of microalgae with complimentary fatty acid profiles might be combined together to optimize the quality of a microalgae diet. Extracting and concentrating proteins and oils from microalgae may also offer advantages for providing optimal nutrition.

## Supporting Information

S1 Fig Absorbance vs. dry weight. Relationship between absorbance (measured by optical density at 540 nm) and dry weight (g∙L^-1^) for all data (*n* = 36).
(TIF)

S2 Fig Nitrate measurement. Example calibration showing relationship between nitrate concentration (0 to 40 μM) and absorbance at 540 nm measured with a microplate reader. Samples from culture fluid were carefully diluted to within the calibrated range.
(TIF)

S3 Fig FAME calibration. Calibration of fatty acid methyl esters derived from *Nannochloropsis* sp. using a Gas Chromatograph. Each of the eight fatty acids types found in *Nannochloropsis* sp. are shown. The C15:0 was added as an internal standard to verify extraction, recovery and transesterification efficiency.
(TIF)

S4 Fig Protein calibration. Calibration of protein concentration using Bovine Serum Albumin (BSA) as the standard using the absorbance measured at 750 nm in a 1cm cuvette.
(TIF)

S5 Fig All growth curves. Growth curves (dry weight accumulation) and nitrate consumption for all cultivation data in the manuscript. Points are the means of three independent replicate cultures and error bars indicate the standard deviation.
(TIF)

S6 Fig Cell size distributions during cultivation. There are *n* = 3 replicate cultures for each time and nutrient treatment.
(TIF)

S1 Table Nutrient medium used in the experiments. (XLSX)

S2 Table Batch cultivation sequence with two photobioreactors. The *Run* is the time at which the pair of photobioreactors were used for cultivation, *Reactor* identifies each of the individual photobioreactors, *Treatment* defines the high-NP or low-NP treatment, *Cultivation time* describes the period of cultivation and *Replicate* indicates each of the three replicate cultivations for any given treatment/ cultivation time combination.
(XLS)

S3 Table Two-way analysis of variance. The effect of time (8, 12, 16 days cultivation) and nutrient treatment (high-NP, low-NP) on the cell diameter of *Nannochloropsis* measured with the Coulter Multisizer 3.
(XLS)
