# Supplementary figures and images for: Production of Fatty Acids and Protein by Nannochloropsis in Flat-Plate Photobioreactors
Source: PLoS One. 2017 Jan 19;12(1):e0170440. doi: 10.1371/journal.pone.0170440 (PMC5245880; doi:10.1371/journal.pone.0170440)

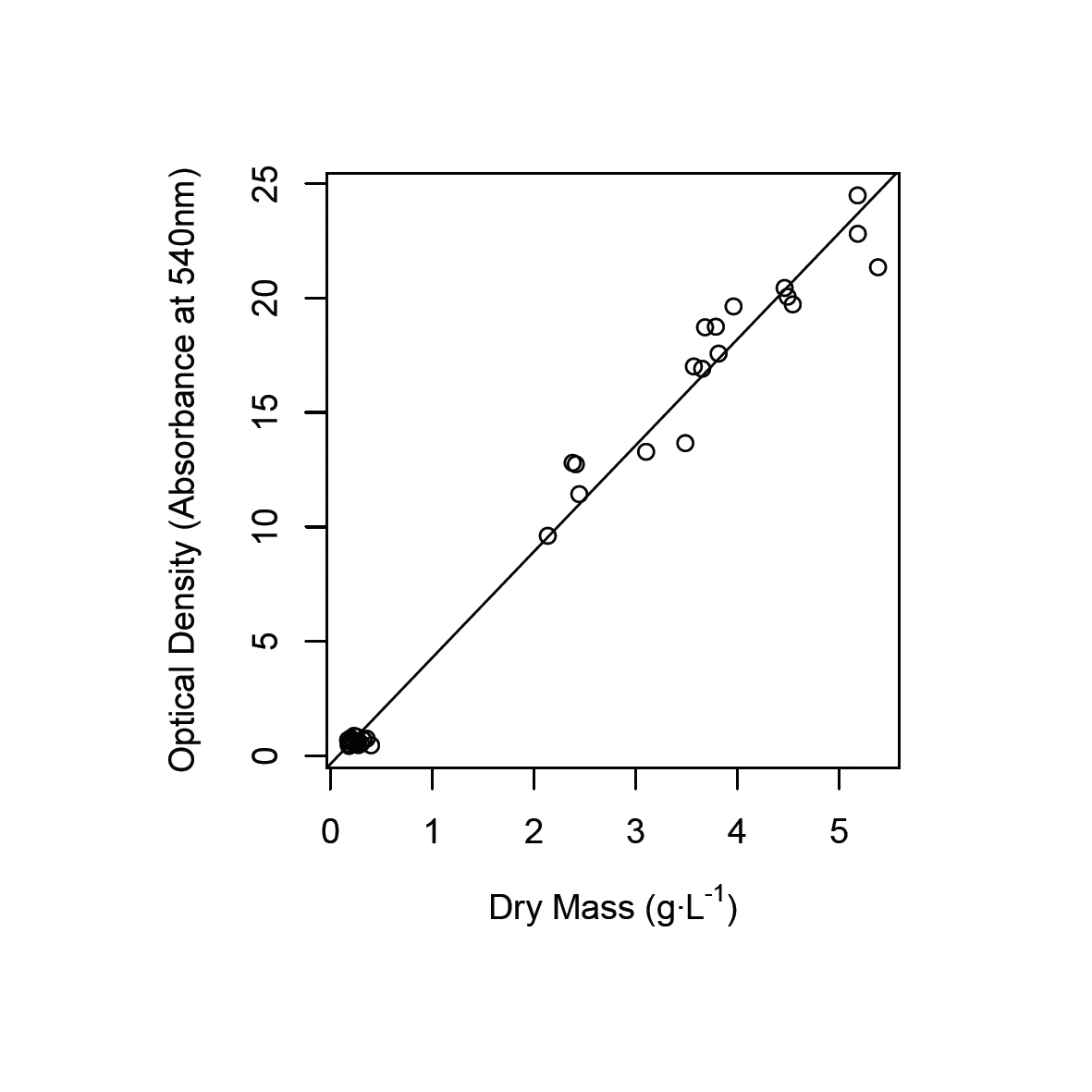

Supplement: S1 Fig — Relationship between absorbance (measured by optical density at 540 nm) and dry weight (g∙L-1) for all data (n = 36). (TIF) [file pone.0170440.s001.tif]

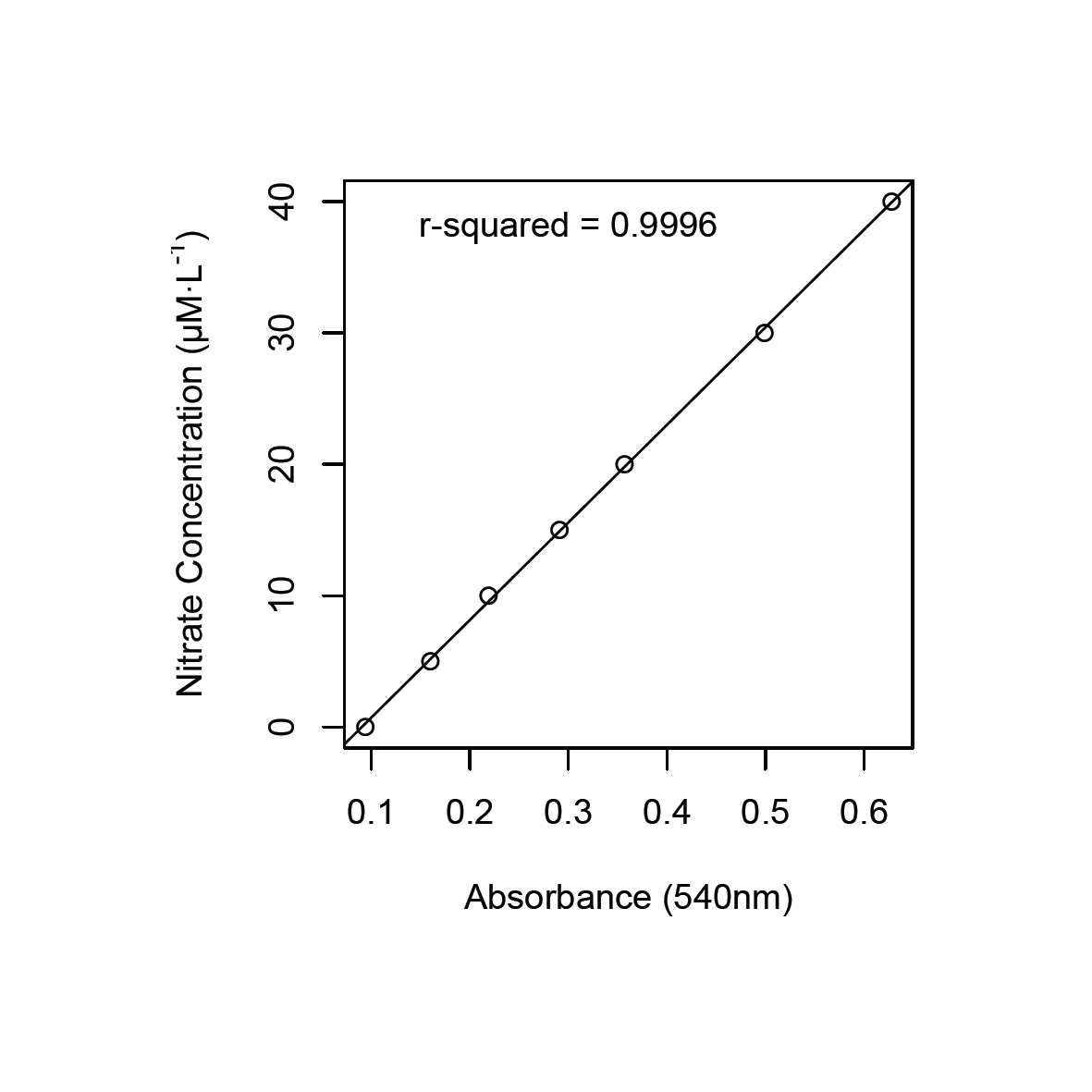

Supplement: S2 Fig — Example calibration showing relationship between nitrate concentration (0 to 40 μM) and absorbance at 540 nm measured with a microplate reader. Samples from culture fluid were carefully diluted to within the calibrated range. (TIF) [file pone.0170440.s002.tif]

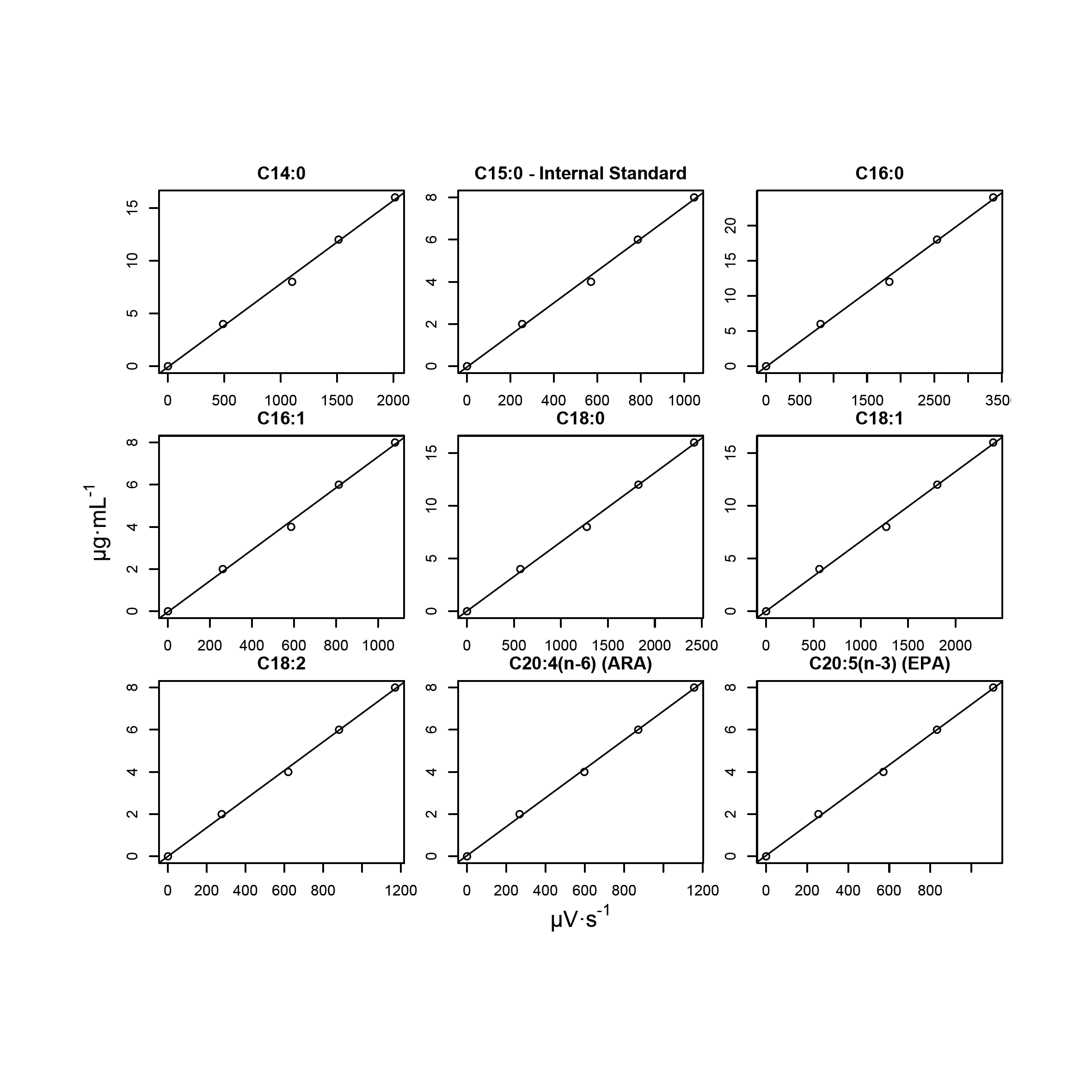

Supplement: S3 Fig — Calibration of fatty acid methyl esters derived from Nannochloropsis sp. using a Gas Chromatograph. Each of the eight fatty acids types found in Nannochloropsis sp. are shown. The C15:0 was added as an internal standard to verify extraction, recovery and transesterification efficiency. (TIF) [file pone.0170440.s003.tif]

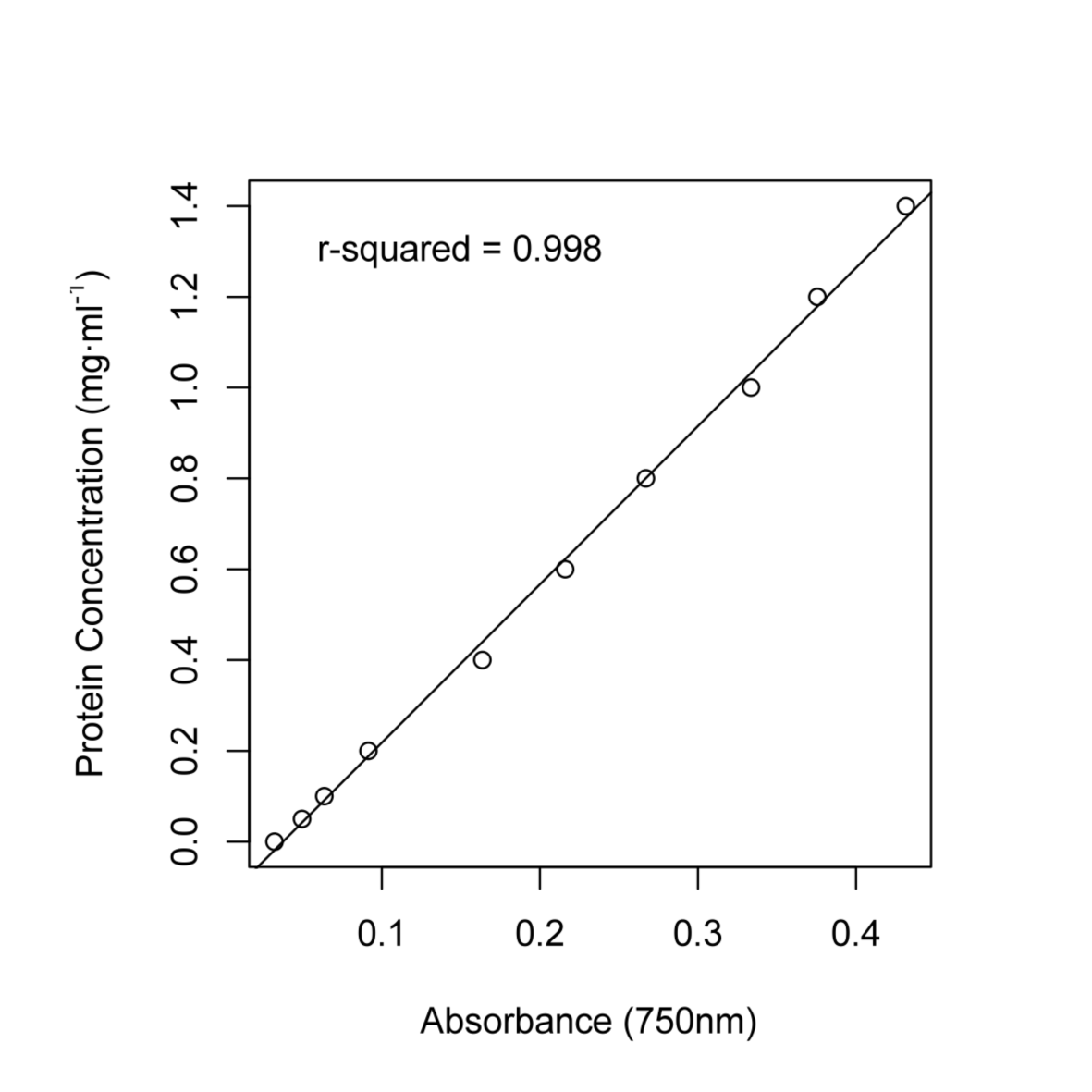

Supplement: S4 Fig — Calibration of protein concentration using Bovine Serum Albumin (BSA) as the standard using the absorbance measured at 750 nm in a 1cm cuvette. (TIF) [file pone.0170440.s004.tif]

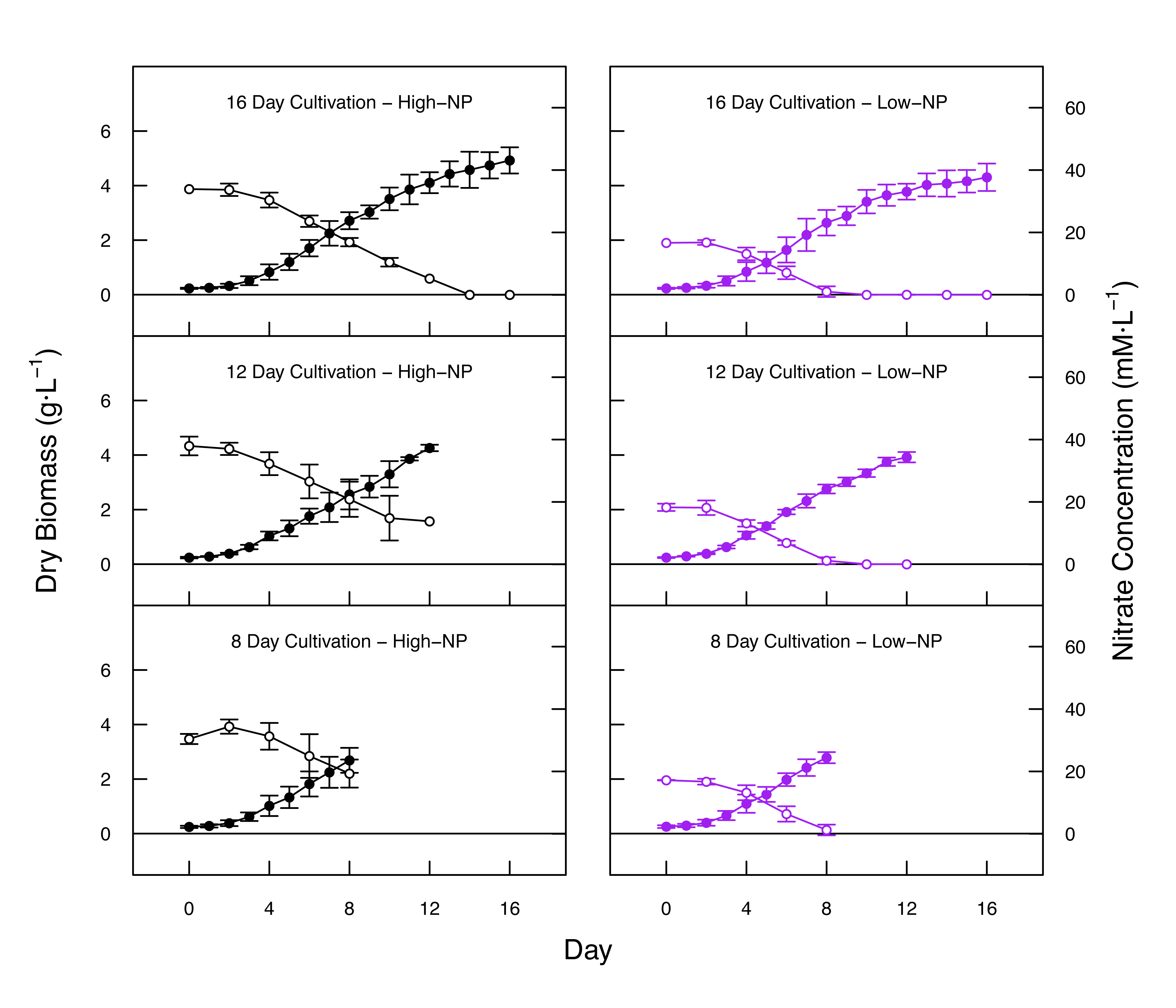

Supplement: S5 Fig — Growth curves (dry weight accumulation) and nitrate consumption for all cultivation data in the manuscript. Points are the means of three independent replicate cultures and error bars indicate the standard deviation. (TIF) [file pone.0170440.s005.tif]

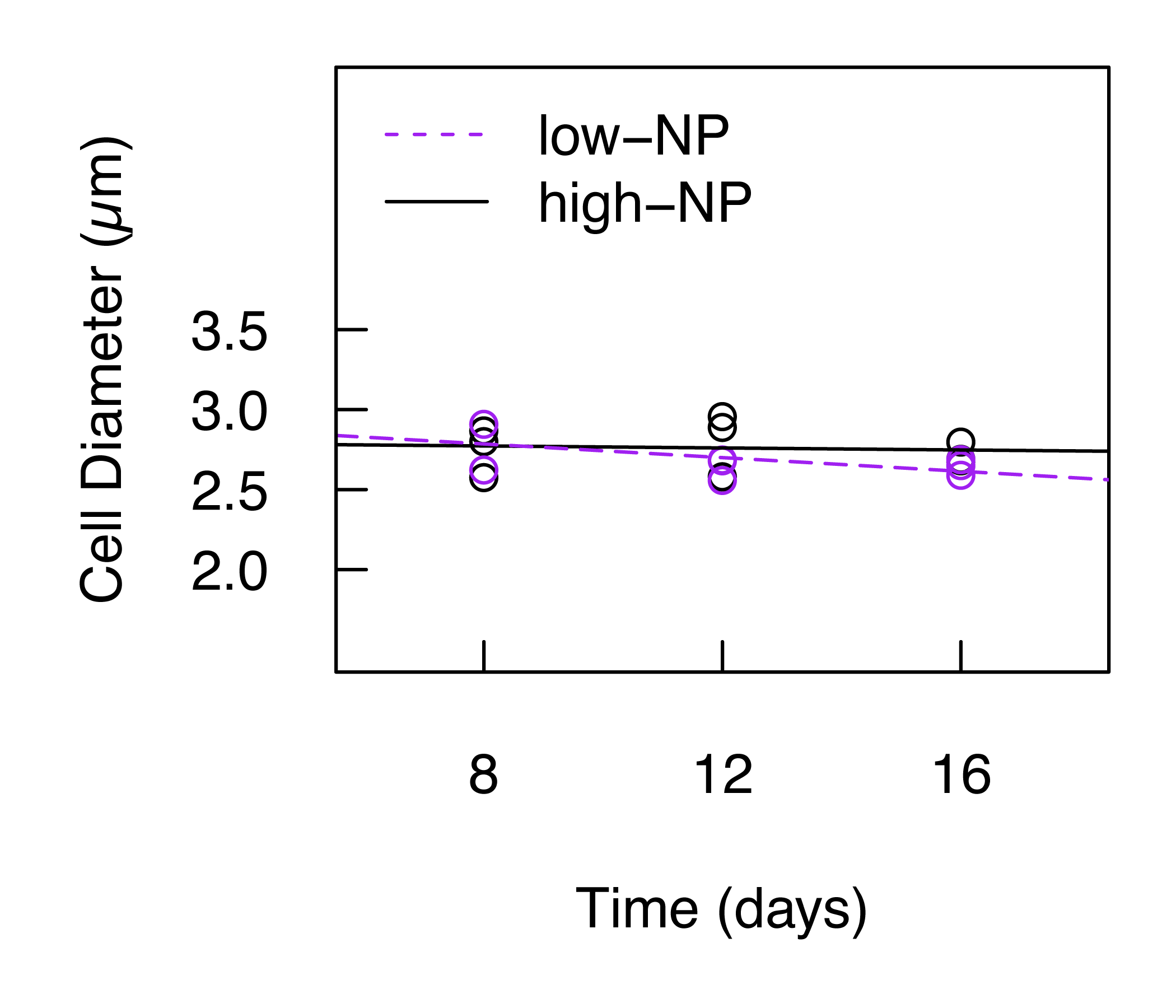

Supplement: S6 Fig — There are n = 3 replicate cultures for each time and nutrient treatment. (TIF) [file pone.0170440.s006.tif]
